# Impacts, Tolerance, Adaptation, and Mitigation of Heat Stress on Wheat under Changing Climates

**DOI:** 10.3390/ijms23052838

**Published:** 2022-03-04

**Authors:** Malu Ram Yadav, Mukesh Choudhary, Jogendra Singh, Milan Kumar Lal, Prakash Kumar Jha, Pushpika Udawat, Narendra Kumar Gupta, Vishnu D. Rajput, Nitin Kumar Garg, Chirag Maheshwari, Muzaffar Hasan, Sunita Gupta, Tarun Kumar Jatwa, Rakesh Kumar, Arvind Kumar Yadav, P. V. Vara Prasad

**Affiliations:** 1 Division of Agronomy, Rajasthan Agricultural Research Institute, Sri Karan Narendra Agriculture University, Jobner, Jaipur 303329, India; mryadav.agro.rari@sknau.ac.in (M.R.Y.); jogendra.pbg.rari@sknau.ac.in (J.S.); nkgupta.pphy.rari@sknau.ac.in (N.K.G.); nkgarg.biochem.rari@sknau.ac.in (N.K.G.); sunita.pphy.rari@sknau.ac.in (S.G.); tkjatwa.ppath@sknau.ac.in (T.K.J.); yadav.arvind580@gmail.com (A.K.Y.); 2 School of Agriculture and Environment, The University of Western Australia, Perth 6009, Australia; mukesh.agri08@gmail.com; 3 Division of Crop Physiology, Biochemistry and Post-Harvest Technology, Indian Council of Agricultural Research (ICAR)-Central Potato Research Institute, Shimla 171001, India; milan.lal@icar.gov.in; 4 Feed the Future Innovation Lab for Collaborative Research on Sustainable Intensification, Kansas State University, Manhattan, KS 66506, USA; vara@ksu.edu; 5 Janardan Rai Nagar Rajasthan Vidyapeeth, Udaipur 313001, India; pushpikka18@gmail.com; 6 Academy of Biology and Biotechnology, Southern Federal University, 344090 Rostov-on-Don, Russia; rajput.vishnu@gmail.com; 7 Division of Biochemistry, Indian Council of Agricultural Research, Indian Agricultural Research Institute, New Delhi 110012, India; cmchandak07@gmail.com; 8 Division of Agro Produce Processing, Central Institute of Agricultural Engineering, Bhopal 462038, India; muzaffarhassan88@gmail.com; 9 Division of Agronomy, Indian Council of Agricultural Research, National Dairy Research Institute, Karnal 132001, India; drdudi_rk@rediffmail.com; 10 Department of Agronomy, Kansas State University, Manhattan, KS 66506, USA

**Keywords:** wheat, climate change, heat stress, heat tolerance, molecular breeding and omics

## Abstract

Heat stress (HS) is one of the major abiotic stresses affecting the production and quality of wheat. Rising temperatures are particularly threatening to wheat production. A detailed overview of morpho-physio-biochemical responses of wheat to HS is critical to identify various tolerance mechanisms and their use in identifying strategies to safeguard wheat production under changing climates. The development of thermotolerant wheat cultivars using conventional or molecular breeding and transgenic approaches is promising. Over the last decade, different omics approaches have revolutionized the way plant breeders and biotechnologists investigate underlying stress tolerance mechanisms and cellular homeostasis. Therefore, developing genomics, transcriptomics, proteomics, and metabolomics data sets and a deeper understanding of HS tolerance mechanisms of different wheat cultivars are needed. The most reliable method to improve plant resilience to HS must include agronomic management strategies, such as the adoption of climate-smart cultivation practices and use of osmoprotectants and cultured soil microbes. However, looking at the complex nature of HS, the adoption of a holistic approach integrating outcomes of breeding, physiological, agronomical, and biotechnological options is required. Our review aims to provide insights concerning morpho-physiological and molecular impacts, tolerance mechanisms, and adaptation strategies of HS in wheat. This review will help scientific communities in the identification, development, and promotion of thermotolerant wheat cultivars and management strategies to minimize negative impacts of HS.

## 1. Introduction

Wheat (*Triticum aestivum* L.) is one of the most widely cultivated cereal crops in the world, making a significant contribution to global cereal production (28%) and trade (41.5%) [1]. About 198 million tonnes of additional wheat grain will be required to feed the increasing human population predicted to be 9.8 billion by 2050 [2]. Wheat production is vulnerable to abiotic and biotic stresses with a stagnant or declining rates of productivity across the globe [3]. Different climatic factors interact spatiotemporally and influence crop growth and production. To better cope with the climatic variables (e.g., temperature and rainfall), their impacts must be quantified and understood. The adverse impact of heat stress (HS) induced through rising ambient temperature and unpredictable climatic variations is clear and threatening wheat production in all ecologies (temperate, subtropical and in tropical) [4,5,6]. 

The mean global temperature of the Earth is expected to increase by 1.5 °C within the next two decades [7]. Recent analysis from scientific communities, including the Goddard Institute for Space Studies (GISS), indicated an increase in average global temperature of 1.04 °C from 1880 to 2019 (NOAA, 2020). This elevated temperature is causing heat stress (HS) that triggers significant changes in the biological and developmental process of wheat, leading to a reduction in grain production [8,9] and grain quality [10]. The optimal temperature requirements of wheat at different growth stages are summarized in Table 1. Wheat is most susceptible to elevated temperature stress especially during anthesis stage and less likely to recover if stressed at this critical stage [11,12,13]. HS can affect the growth and development of wheat through alteration of physio-bio-chemical processes, such as photosynthesis, respiration, oxidative damage, activity of stress-induced hormones, proteins and anti-oxidative enzymes, water and nutrient relations, and yield forming attributes (biomass, tiller count, grain number and size) upon exposure to temperatures above the optimum range [14,15,16,17,18,19]. 

HS impacts the wheat plant in both indirect and direct ways. Indirect injury includes poor seed germination, decreased growth, enhancement in leaf senescence, and a reduction in photosynthesis and floret fertility [12,13,21], whereas direct injuries include protein denaturation, increased fluidity of membrane lipids, and aggregation of proteins [18,19]. However, the consequence of HS depends on the duration and intensity of stress, or genotypes [2,21]. Therefore, emphasis should be placed on sustaining the wheat yields through the identification of tolerant genotypes and promotion of breeding strategies and management practices that can help to build HS resilience and safeguard the wheat production from HS [22]. An improved understanding of morphological and physiological traits associated with HS tolerance has pragmatic implications for devising countermeasures, e.g., to identify various tolerance mechanisms and their use in alleviation strategies [23,24,25]. The mapping of genomic regions governing such physiological traits helped to identify genes (or QTLs) conferring HS tolerance, which serve as a strong base for the marker-assisted (MAS) breeding of HS tolerance in wheat [24,26]. Conventional breeding approaches (screening and selection of germplasm) and molecular breeding are promising genetic strategies for the development of HS tolerant wheat genotypes and the introduction of these cultivars in the non-conventional area will help to overcome the issue of food and nutritional security [2,27]. 

Besides, different biotechnological tools, e.g., transgenics and gene editing, along with recently developed omics approaches can help to develop HS tolerant cultivars [28]. The most reliable and inexpensive method to improve plant resilience to HS includes a combination of stress tolerant genotypes and agronomic management strategies, such as the application of exogenous protectants, adoption of climate-smart cultivation practices including conservation agriculture (CA), micro-irrigation and mulching, and use of cultured soil microbes [6,29,30,31,32]. However, the limited progress and success in the development of HS resilience in wheat can be attributed to the lack of coordinated efforts by plant breeders, biotechnologists, agronomists, and physiologists. Therefore, the adoption of a holistic multidisciplinary approach integrating outcomes of breeding, physiological, biotechnological, and agronomical options is needed for providing practical solutions. In this review, we have emphasized and synthesized the impact of HS on various wheat physio-biochemical and molecular processes and discussed conventional and non-conventional breeding technologies with agronomic management aspects on how to enhance HS tolerance in wheat under current and future changing climates.

## 2. Responses of Wheat to HS

### 2.1. Morphological and Phenological Responses

**Figure 1:**
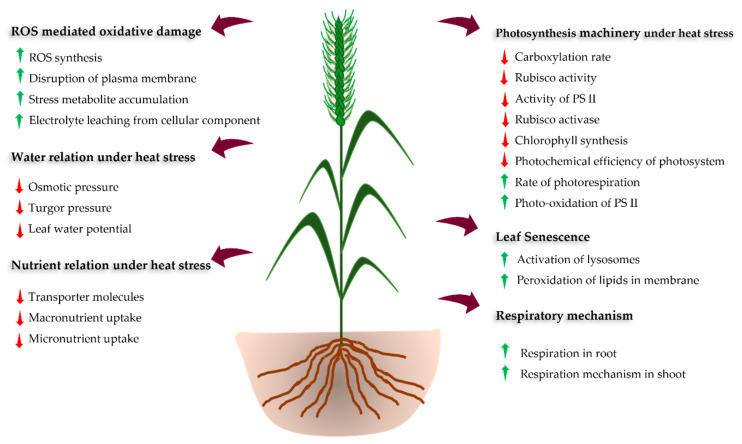
Schematic diagram showing impacts and responses of plants to heat stress.

HS affects diverse morpho-phenological stages, such as germination, seedling emergence, tillering, floral initiation, pollination, fertilization, and ultimately yield and grain quality [11,12,14] (Figure 1 and Table 2). However, the impact of HS on these phenological stages depends upon the magnitude and length of exposure, genotypes, soil moisture status, and concentration of elevated carbon in the atmosphere [21,33]. The HS at the early seedling stage leads to poor seedling establishment. At the early vegetative stage of the crop, HS reduces root and shoot growth reducing the green leaf area and the number of effective tillers per plant [34,35]. Prolonged exposure to elevated temperature leads to cell injuries and the gradual shedding of leaves and abortion of flower and fruits [35,36]. during and just after anthesis (within 10 days) leads to flower abortion and thus reducing grain number, grain filling and maturity period [11,37,38]. The HS during the initiation of flowering to early grain filling stages (terminal HS) is more severe, causing a drastic reduction in the dry matter accumulation and grain quality of wheat. Therefore, more emphasis should be placed upon the management of terminal HS to sustain the wheat yields [24,39]. 

### 2.2. Physiological and Molecular Responses

Most of the physiological functions in plants are temperature dependent and any deviation over the optimum temperature hampers the growth and development of plants (Table 2). HS reduces photosynthetic efficiency which eventually affects the plant growth and biomass production [22,56]. The reduction in photosynthesis rate under HS is correlated with the increase in the non-photorespiratory processes and reduction of soluble proteins, Rubisco and Rubisco binding proteins [72,73]. These sequential changes results in reduced leaf area, less effective photosynthetic machinery, early leaf senescence, overproduction of reactive oxygen species (ROS), disruption of thylakoid membrane, alteration of enzyme action, and denaturation of heat shock proteins (HSPs), which ultimately reduces wheat productivity [6,55,74]. The reduction in total chlorophyll content with accelerated leaf senescence diminishes the photosynthetic capacity of the plant [61]. Furthermore, respiration and photorespiration rate increase with temperature above the threshold level, resulting in the reduction of availability and transport of photoassimilates from leaves to the grain. This hampers the growth and grain filling process [75,76]. 

Water status in the plant is crucial especially under HS as the temperature of plant tissue (canopy temperature) can be optimized by water uptake and transpiration. Thus, leaf relative water content (LRWC), leaf water potential (LWP), rate of transpiration, and stomatal conductance are strongly influenced by canopy temperature (Table 2) [59]. Higher vapor pressure deficits under HS lead to a higher evapotranspiration rate which ultimately decreases LWRC and LWP (Table 2). However, limited information is available about the effect of HS in relation to the nutrient status of the crop [77]. The uptake and translocation of nitrogen (N) in wheat declined under HS due to a reduction in nitrate reductase activity (NRA) in plants [62]. Phosphorus (P), potassium (K), sulphur (S), and sodium (Na) helps to maintain the redox state of the cell and hence protects the cell membranes, improving the antioxidant defense system and osmotic potential, which ultimately enhances the photosynthetic rate. These nutrients reduce the activity of nicotinamide adenine dinucleotide phosphate (NADPH) oxidases and retain the photosynthetic electron transport activity, which helps to reduce ROS thus maintain the redox state of the cell under HS. However, reduced root growth under HS reduces uptake, assimilation, and translocation of most of the nutrients [78].

HS triggers the production of ROS, such as super-oxides (O^2−^), hydroxyl radicals (OH^−^), and hydrogen peroxide (H_2_O_2_), resulting in severe oxidative damage to the plant [79]. HS can also damage the plasma, mitochondrial, and chloroplast membranes due to lipid peroxidation and concomitant H_2_O_2_ production [80]. To the eliminate the accumulated ROS and maintain metabolic activities and productivity [81], the plant has a well-organized cellular stress-response system (defense systems) composed mainly of transcription factors (TFs) and HSPs [82]. Plants possess signal transduction molecules (Calcium-dependent protein kinases (CDPKs), mitogen-activated protein kinase (MAPKs), sugar (as signalling molecule), and phytohormones) that act as an activator of the stress-responsive gene during HS [68,83]. For example, dehydration-responsive element-binding protein (DREB) is a signal transduction molecule that activates the stress-responsive genes through stress signaling induced expression in wheat. These signal transduction molecules together with TFs activate stress-responsive genes [84,85]. For example, *TaHSFA6f* gene has been reported to function in sensing the HS and overexpression of this gene imparted heat tolerance in wheat [86].

### 2.3. Heat Stress and Sensitive Stages of Wheat 

The impact of HS is more severe during reproductive stages in comparison to the vegetative phase [11,12,52]. HS hinders pollen tube growth, resulting in reduced germination of pollen grains on stigma and impacts embryo formation [12]. Translocation of assimilates and the rate and duration of grain filling are also directly affected by elevated temperature [87]. Exposure to high temperature stress during the division of pollen mother cells can reduce grain setting [11,12,88]. High temperature (30–38 °C) during the reproductive phase can lead to the reduction of the total biomass of wheat up to 44% [48]. HS during the early grain filling stage declines the synthesis of starch in the wheat grain but increases the concentration of total soluble sugars (Table 2) [89]. Although the protein content increases under HS, the functionality of proteins significantly decreases, adversely affecting its end-use [90]. Even after a high percentage of grain protein, total protein yield reduces under HS due to a drastic reduction in the grain yield [91]. However, an increased level of protein content has been reported to reduce the sedimentation index, which affects the bread-making quality of wheat flour [92,93]. The heat-inducible accumulation of gliadins under HS also deteriorates the flour quality of wheat [94].

Temperature is the most important factor influencing the growth and development of wheat (Table 1). The growth and development start with seed germination, which requires optimum temperature and moisture. HS is an important key abiotic stress that severely affects the seedling establishment of wheat [95,96]. The germination index and germination potential of two wheat cultivars, namely DBA Aurora and L6, were significantly reduced under HS conditions [97]. The reduction in the germination percentage and seedling establishment under heat stress conditions might be due to a reduction in the antioxidant system, production of ROS, increased lipid peroxidation, and differential expression of miRNAs [16]. Recent studies on seedling establishment have revealed that the expression of miRNAs and epigenetic changes in wheat seedlings might be key factors that help to optimize the seed vigor for superior breeding of germplasm and varieties under heat stress [98]. The seedling with high vigor is projected to be more effective in radiation and resource use efficiency. High seedling vigor has no transgenerational effect on the vegetative and reproductive phase [99]. During the vegetative growth of wheat, the tissues exposed to HS leak electrolytes, damaging leaf members, and decreasing photosynthesis [100,101]. HS severely impacts on photosystem II and thylakoid membranes, which are most sensitive cell structures [102]. 

HS stress is a severe threat to wheat production when it occurs during reproductive and grain-filling phases (Table 2) [11,12,13,14,55]. Wheat crops experience HS in different phenological stages. However, the impact of HS during the reproductive phase is relatively more detrimental as compared to the vegetative phase which ultimately affects the grain quality and yield (Table 2). HS during reproductive stages of crop development decreases grain yield up to 30% [11,19,103]. The optimum temperature for anthesis and grain filling stage was suggested to be in the range of 12 °C (night-time minimum)–24 °C (daytime maximum), and 8–6 days before anthesis stages are identified to be the most sensitive stages to HS [11,19]. Likewise, results of 30 wheat crop models from different locations around the globe where mean temperatures in the growing season ranged from 15 to 32 °C with artificial heating indicated that grain yield of wheat started to decline at a majority of the wheat-growing locations [104]. The simulated median temperature impact on declining wheat yield ranged between 1% and 28% for an increase in temperature of 2 °C, and this value rose to between 6% and 55% for a temperature of 4 °C [105]. 

Moreover, the reduction in the number of spikelets per panicle, hundred-grain weight, and seed setting rate was reported in wheat under HS [2,12,19,106] (Table 2). Pollen tube development after pollination is extremely sensitive to heat stress, leading to abnormal ovary development, ultimately resulting in lower yield as well as quality of grains [104,107,108]. Harvest index (HI) and its correlation with heat stress is most significant in wheat among all cereals. HI plummets when daily mean temperatures cross 16 °C and a significant loss of yield occurs when it crosses 30 °C [12]. The impact is more severe when there is higher night temperature, resulting in reduced grain yield, attributed primarily to the reduced spike number [14,109,110]. HS induced through high night temperatures, especially during the grain filling stage, can reduce grain yield, HI, 1000-grain weight, and grain weight per spike [111]. These studies provide key traits for wheat breeders to enhance high night temperature tolerance in wheat.

## 3. Heat Tolerance Mechanisms

Plants as sessile organisms are prone to encounter various unfavorable conditions (environmental stresses). Plants have developed numerous protective/adaptive mechanisms both at physiological and molecular levels to cope with stress. These include short term avoidance/acclimation mechanism (leaf orientation, transpirational cooling, and changes in membrane lipid composition) or long-term evolutionary tolerance mechanisms, including ROS scavenging antioxidant systems (Table 3) [112,113,114]. These stress-responsive mechanisms help to protect and repair the damaged proteins and membranes and thus impart HS tolerance in plants [115,116].

### 3.1. Antioxidant Defense System

ROS are produced in limited amounts by different cell organelles (mitochondria, chloroplast, and peroxisome) and the cell membrane (Table 3) and are needed in various metabolic pathways, such as lignin formation, leaf and flower abscission, cell senescence, and ripening of fruits [117]. However, HS results in the over-production of ROS causing severe damage to chlorophyll, DNA, protein, and membranes [79]. Therefore, accumulated ROS need to be detoxified to safeguard the plant integrity and development [70,71]. Antioxidant defense systems in plants include both enzymatic as well as non-enzymatic systems. The enzymatic system includes ascorbate peroxidase (APX), catalase (CAT), glutathione reductase (GR), polyphenol oxidase (PO), peroxidase and superoxide dismutase (SOD), whereas the non-enzymatic system includes carotenes, ascorbic acid (vitamin C), α-tocopherol, and more. These antioxidants get activated on sensing the HS and protect cells by regulating the oxidative damage [118]. The SOD scavenges the superoxide free radicle (O_2_^−^) and converts it into H_2_O_2_. Thereafter, CAT and APX convert H_2_O_2_ into water (H_2_O).

### 3.2. Heat Shock Proteins (HSPs)

HS stimulates the expression of heat shock genes (HSGs) that encode the HSPs [119]. The HSPs are water-soluble and consequently imparts HS tolerance in plants probably via hydrating cellular structures. In plants, five well-characterized families of HSPs are: HSP20, HSP60, HSP70, HSP90, and HSP100 [120]. One of the characteristic functions of HSP20 is the degradation of some proteins that have unsuitable folding. HSP60 and HSP70 are the most important conserved proteins and consistent compounds to combat HS [121]. HSGs have a heat shock element (HSE) at their promoter site which triggers the transcription of HSG. HSE consist of a palindromic nucleotide sequence that is recognized by heat shock transcriptional factor or heat shock factor (HSF) for binding to the promoter. HSFs are primarily expressed in normal conditions, and present in monomeric form, bound to HSP70 in the cytoplasm. When the plant senses the heat shock, the monomers of HSFs separate from the HSP70 and enter the nucleus. In the nucleus, these monomers of HSFs form trimer that binds to HSEs and result in inducing the expression of HSG thus imparting thermotolerance [16]. HSP90 has been reported to impart heat tolerance at the seedling stage [2]. One unique function of this class is the reactivation of aggregated proteins by the re-solubilization of non-functional protein aggregates [121]. HSP90 with other classes (HSP70 and HSP60) has an important role in signalling protein function and trafficking during HS [16]. HSP 100, also known as *ClpB* (caseinolytic protease B proteins), assists in the disaggregation mechanism, i.e., unfold the misfolded polypeptides (due to heat stress), hence supporting other HSPs, e.g., sHSP and HSP70, in the proper folding of proteins [121]. Overall, HSPs plays a significant role in the maturation of protein complexes and the degradation of damaged or misfolded peptides, regulating the activity of many signal transduction proteins during HS.

### 3.3. Delayed Leaf Senescence/Stay Green 

Delayed senescence/stay green, being a critical trait for genetic improvement, has a pragmatic role in abiotic stress tolerance in plants. Rate of senescence is determined by the rate at which chlorophyll degrades. This in turn hampers the photosynthesis and ultimately results in reduced yields. There are two types of stay green traits: (1) functional traits are of agronomic interest whereas the photosynthetic capacity of plants is maintained compared with standard genotypes either through delaying the onset of senescence (type-A) or through slowing the rate of senescence is (type-B); and (2) non-functional/cosmetic traits are those in which senescence occurs at normal rates with a decline in photosynthetic activity, but leaf color is retained due to the failure of the chlorophyll (Chl) degradation pathway (type-C) [122]. Several crops have been evaluated for the delayed senescence/stay green characteristic, but successful breeding efforts for this trait are mostly confined to few crops, including wheat and sorghum. For example, TaNAM (encoding a NAC-type transcription factor (TF)) RNAi line with impaired nutrient remobilization from leaves facilitate a delay in senescence in wheat cultivars [123]. The significant positive association has been assessed among high grain yield in wheat genotypes exhibiting delayed senescence/stay green characteristic [124]. A strong positive correlation between stay green traits (Chl content and flag leaf area) and yield enhancing characters (grain filling rates and total grain weight) was observed in “Dena” and “Vee/Nac” genotypes of wheat, attributed to the improved photosynthetic activity under HS exposure [125]. A stay green QTL (44 loci) of “Seri/Babax” wheat mapping population raised under HS environment showed a strong positive association with HS tolerance, grain yield, and grain filling rates [126]. Overall, delayed senescence/stay green traits are promising to identify and develop thermotolerant wheat cultivars, thus having the potential to sustain wheat productivity under the threat of HS.

### 3.4. Canopy Temperature Depression (CTD)

CTD is the difference between canopy temperature and air temperature, and under well irrigated wheat plants can transpire and keep their canopies cooler. Vapor pressure deficit has a large effect on CTD, while net radiation, air temperature, and wind speed have slight effects [127]. CTD effected by biological and environmental factors, e.g., water status of soil, wind, evapotranspiration, cloudiness, conduction systems, plant metabolism, air temperature, relative humidity, and continuous radiation [128], has preferably been measured in high air temperature and low relative humidity because of high vapor pressure deficit conditions [129]. At the end of 1980, CIMMYT began CTD measurements on different irrigated experiments in Northwest Mexico. Phenotypic correlations of CTD with grain yield were occasionally positive [130,131]. CTD has been used as a selection criterion for tolerance to drought and high temperature stress in wheat breeding and the used breeding method is generally mass selection in early generations like F3. According to this method, firstly, bulks that show a high CTD value (have cool canopy) are selected in the F3 generation. Later, single plants are selected that show high stomata conductance (g) among bulks that show cool canopy at the same selection generation. Thus, both of these traits are used in the same breeding program [128]. Cool canopy during grain filling period in wheat is an important physiological principle for high temperature stress tolerance [132]. CTD used as selection criteria for heat tolerance was recorded at 12 h, 14 h, and 16 h at 7-day intervals on bright sunny days. Correlation study showed that CTD also displayed a significant correlation with yield traits, e.g., GFD (*r* = 0.78), grain yield (*r* = 0.84), and biomass (*r* = 0.81) [124].

### 3.5. Acquired Thermo-Tolerance and Temperature Sensing and Signaling

Acquired thermotolerance is a condition where a plant acquires tolerance against excessive HS after a short exposure to sub-lethal temperature. The plasma membrane serves as first sensor for plant cells for early sensing of mild temperature increments which in turn activates the transient opening and depolarization of specific heat sensitive Ca^2+^ channels [133]. Cyclic nucleotide gates channels (CNGCs), a protein encoded by CNGC genes, were identified as the primary heat-sensors in plants [134]. In nature, over time, plants experience a gradual rise in temperature from sub-lethal to lethal. During the temperature rise, several physiological and molecular changes occur that help plants to acquire thermotolerance. Studies conducted on *Arabidopsis* mutants indicated that, other than HSP (e.g., HSP32 and HSP101), various other pathways, such as abscisic acid (ABA), ROS, and salicyclic acid (SA), also impart acquired thermotolerance [135]. The inherent ability of plant tissues to respond to HS by transient reprogramming of gene expression pattern is defined as heat shock response (HSR). Although the optimum growth temperature varies among genus and species, beyond the optimum temperature range, all plants exhibit HSR [134]. HSR includes synthesis of different HSPs accompanied with resumption of normal protein synthesis. The transient synthesis of HSPs under long-term HS indicates three possible responses of signal triggering, namely the response is either lost, inactivated, or no longer recognized [136]. However, detecting the direct role of HSPs in enhancing thermotolerance is difficult due to the complicated nature of HS tolerance. Plant growth promoting rhizobacteria (PGPR) have also been found to improve the thermo-tolerance in wheat through multiple cellular responses, including low production of ROS, reduced membrane damage, and maintenance of chloroplast structure and function, redox enzymes, and osmolytes [137]. Acquired thermo-tolerance is a crucial strategy for crops to cope with lethal HS via reprogramming of gene expression. 

The effective survival response of a plant to a stress tolerance mechanism involves two key factors, first the timely perception of stress and second the cascade of signal transduction. Two-way genetic analysis in combination with gene expression studies uncovered many signaling pathways and their components [138]. A number of perception and signaling mechanisms are utilized by plants for various stresses, although some are highly specific to one kind of stress, while others are mainly involved in crosstalk. The cell redox system which maintains a balance between oxidant and the antioxidant pool is a key player in stress signaling. A number of studies confirmed that chemical signaling cascades, such as ROS, Ca^2+^, and plant hormones, are activated through reprogramming at the genome level [70]. Physical state transition in the membrane, induced by temperature variation, plays an important role in sensing, influencing gene expression. HS causes various changes at the membrane level, such as a change in the ratio of saturated to unsaturated fatty acid and thylakoid membrane rigidification. These changes trigger HSP which ultimately invokes the altered expression profiles of enzymes, indicating the critical role of the membrane in sensing high temperature [139]. Sung et al. [140] reviewed the role of the thylakoid membrane as a heat sensor due to the presence of high unsaturated fatty acids and temperature-sensitive photosystems. The Ca^2+^ ion ubiquitously plays the role of the intracellular secondary messenger in different signaling pathways. Ca^2+^ ion influx and the CDPK together regulate HSPs expression [65], thus play a critical role in temperature sensing and signaling under elevated temperature. 

## 4. Adaptation Strategies

The development of heat-tolerant genotypes of wheat along with the adoption of improved agronomic practices are required to sustain wheat productivity under the forecasted climate change scenario. Recent advances in breeding as well as biotechnological and agronomic strategies for improving heat tolerance, thus harvesting better yields of wheat under HS environments, are discussed below.

### 4.1. Genetic Approaches

**Figure 2:**
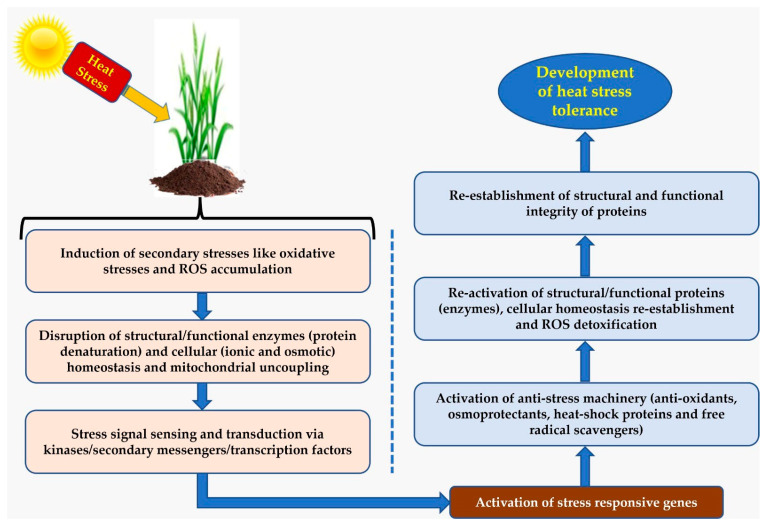
Development of heat tolerance responses and mechanism under HS in wheat.

Developing HS tolerant wheat cultivars is an efficient and viable approach for tackling the rising temperature in wheat production ecology [27]. The initial step of breeding for HS resistance involves screening under testing environments followed by the selection of relatively HS tolerant cultivars (Figure 2). 

The selected cultivars can be used to develop HS tolerant varieties. In past, more emphasis was placed on yield improvement and efforts lacked for the identification of favorable alleles in wheat germplasm for HS tolerance [2,141]. However, realizing the impacts of climate change, the focus has now shifted to the development of heat-tolerant wheat genotypes [124]. With the emergence of cheaper platforms for genotyping, molecular breeding emerged at a rapid pace for developing climate-smart genotypes. Besides that, the development of transgenic wheat cultivars also holds promise for the sustainable production of wheat. However, the integration of conventional and genomics assisted breeding techniques along with use of advanced high throughput phenotyping platforms can only serve as an efficient approach for the development of HS tolerant wheat cultivars.

#### 4.1.1. Conventional Breeding Approach 

Conventional breeding relies upon the extensive phenotypic characterization of the germplasm under specific stress environments. The availability of genetic diversity for trait of interest is an important criterion to start with for HS breeding. Although some studies in wheat advocated the presence of genetic diversity for HS tolerance but up to a limited extent [59]. This is due to the use of limited germplasm for breeding modern cultivars [142]. However, wild ancestors possess an enormous amount of genetic variation for HS tolerance associated traits as compared to modern cultivars [141]. Landraces are HS tolerant due to the high amount of leaf chlorophyll contents and higher conductance. In the recent past, the focus of wheat breeders has shifted to the utilization of landraces for the derivation of HS resistance varieties [143]. Some wild relatives of wheat, i.e., *Aegilops speltoides*, *Ae. Longissimi*, *Ae. Searsii, Triticum dicoccoides*, and *T. monococcum*, have been identified as a promising source for tolerating high temperature in wheat [144]. Pre-breeding is the process of utilization of wild ancestors to diversify the genetic architecture of existing germplasm for several traits. Pre-breeding can be carried out to introgress the heat resistance-associated traits into bread wheat [59]. However, the progress in pre-breeding through conventional techniques has been limited owing to the slow pace of progress and problem of linkage drag [145].

Selection can be effective for the highly heritable traits that are positively correlated with optimum grain yields under HS (Figure 2) [8,60]. However, yield contributing traits are polygenic in nature and have relatively low heritability, making their direct selection difficult. In such cases, the indicator traits (proxy traits) that exhibit better correlation with wheat grain yield under HS, *viz.* canopy temperature depression, rate of photosynthesis, stomatal conductance of flag leaf, membrane thermo-stability, and chlorophyll fluorescence can be used [146]. The growth and phenological traits for HS tolerance include rapid ground coverage (biomass) [147], rolling of leaf [148], early flowering, and heat susceptibility index (HSI) [149]. HSI is an index derived from different traits (yield, crop height, spike length and others) that indicates the relative tolerance of a genotype and the total reduction in grain yield or other traits in response to heat stress. The lower HSI values (<1.0) for a trait indicate better thermal tolerance and high resilience and plasticity for high temperatures. This helps in the categorization of wheat genotypes into susceptible, tolerant, and highly tolerant to HS. The genotypes with low HIS values can be classified as heat stress tolerant, whereas those with high values can be grouped into heat sensitive groups. Physiological traits associated with HS resistance include spike photosynthesis, photosynthesis rate, leaf chlorophyll content, canopy temperature depression (CTD), membrane thermo-stability, flag leaf stomatal conductance, stay-green duration, stem reserves, stem carbohydrate remobilization, pollen viability, and antioxidant activity [150,151]. Early heading wheat varieties is desired to cope with terminal HS as they complete their life cycle under optimal temperature conditions. Stay green character is an important trait for the rapid screening of a large number of lines using a chlorophyll meter or visual rating, but the trait may sometimes hinder with remobilization of stem reserves. Hence, in addition to stay green trait, membrane stability (electrolyte leakage) and the reduction of tetrazolium triphenyl chloride in mitochondria (spectrophotometer) have been suggested as a quick method for the screening and identification of HS tolerant cultivars [2]. Further, optimum grain yield under HS (grain filling) also serves as an important selection parameter. Although the first green revolution resulted from the exploitation of shoot architecture by the selection for the semi-dwarf trait in rice and wheat, to address HS and drought stress, root architecture needs to be explored for obtaining optimum yields [28]. The root architecture for HS tolerance exhibits the most compact and uniform, growth with maximum lateral root extension from the stem base and capable of producing greater length of roots in the deeper soil layers. Deeper roots support better soil moisture extraction, which leads to transpirational cooling and canopy temperature depression, which helps in minimizing impact of HS. Dynamic plasticity of root architecture in response to stress makes it a strong candidate for developing stress-resilient cultivars. Hence, the traits associated with favourable root architecture under HS are crucial.

HS tolerance is a complex trait and hence rapid gains can be achieved through the development and use of selection indices as selection criteria. HSI has been reported as a reliable selection criterion for the selection of HS tolerant genotypes in wheat, with values ≤0.5 indicating relatively greater heat stress tolerance [141]. Recently, heat sensitivity indices have also been developed using thousand-grain weight and duration of grain filling. Hence, selection indices can help to select stable genotypes that perform equally well under stress and optimal environments [8].

#### 4.1.2. Molecular Breeding

The limitation of traditional breeding approaches lies in the investment of more time. These limitations can be overcome by the use of molecular approaches that serve as an efficient tool for assisting climate-resilient breeding [152]. Simple sequence repeats (SSRs) served as ideal markers for diversity assessment as well as QTL mapping due to their relatively high abundance, uniform distribution across the genome, co-dominant nature, and high polymorphism. However, in the recent past, there has been a gradual shift from SSRs to single nucleotide polymorphisms (SNPs) due to the emergence of cheaper sequencing platforms (Table 4).

#### 4.1.3. QTL Analysis 

QTL analysis is the strategy for the molecular markers-based identification of genomic regions governing the significant amount of variance for the target trait. QTL analysis for HS tolerance associated traits uses a segregating population known as mapping population, which is developed using parental lines exhibiting contrast for HS tolerance associated traits. Hence, the screening of germplasm provides the raw material for QTL analysis, *viz*. HS tolerant and susceptible parents. QTL analysis led to rapid progress in the mapping of major as well as minor genomic regions imparting HS tolerance [161]. QTL mapping for HS tolerance in wheat was initiated in 1991 with the use of Langdon chromosome substitution lines that resulted in the identification of HS tolerant genes on chromosomes 3A and 3B. Later, in another QTL mapping study, the same chromosomes were found to carry the genes for HS tolerance conferred by wheat cultivar (cv) Hope [162]. Later, numerous QTLs for HS associated traits were mapped in wheat (Table 4). However, most QTLs are of minor effect and large genomic intervals spanning those QTLs make their direct transfer a difficult task. However, a few major QTLs have also been identified (Table 4). Major QTLs can be easily transferred and can also be further targeted for the identification of candidate genes through fine mapping [163].

For the identification of QTLs, SNP based study for mapping HS tolerance associated traits, namely thylakoid and plasma membrane damage, and chlorophyll content was carried out in wheat. The study revealed the presence of two major QTLs for thylakoid membrane damage (*QHttmd.ksu-7A*) and SPAD chlorophyll content (*QHtscc.ksu-7A*) on 7A exhibiting phenotypic variation of over 30% [158]. Chlorophyll fluorescence kinetics (CFKs) is also a good indicator of HS resistance. Considering this, mapping studies in a double haploid (DH) population led to the identification of a major QTL, *QFm.cgb-4A*, for maximum fluorescence, conferred by Hanxuan10. Such major QTLs will assist in unravelling the molecular basis of chlorophyll fluorescence kinetics [51]. Furthermore, Sharma et al. (2017) used a non-destructive approach to identify three major QTLs explaining over 20% of phenotypic variance for the maximum quantum efficiency of photosystem II (Fv/Fm ratio) [164]. Along with the earlier identified major QTLs, recently identified major QTLs for grain yield per plant, *QGYPHS1*, thousand kernel weight, *QTGW-2A.1*, and grain yield, *QYld.aww-1B.2*, can be targeted for introgression in HS tolerance breeding programs in wheat [165]. However, prior to the introgression of QTLs, validation of the identified candidate genes or QTLs is a critical step as it helps in the identification and avoidance of linkage drag effects associated with yield reductions or other undesirable effects.

#### 4.1.4. Marker-Assisted Selection (MAS)

Numerous QTL analysis studies in wheat revealed many minor QTLs, but few major QTLs, as HS tolerance is a complex mechanism. The next step under HS resistance breeding is the introgression of identified QTLs in elite cultivars through a simple and quick approach. MAS is a rapid technique of crop improvement as it helps to detect (follow) the target genes (QTLs) in lines, cultivars, and breeding populations through indirect selection (via linked markers) at the early stage of crop irrespective of the target environment [166]. MAS based approaches include marker-assisted backcrossing (MABC), marker-assisted recurrent selection (MARS), genome wide association studies (GWAS) and genomic selection (GS). MABC and MARS are the major molecular breeding approaches that have been successfully utilized to a larger extent for developing climate-resilient cultivars in maize [167]. The major QTLs for HS resistance associated traits can be introgressed via MABC [168], while favorable minor and major effects of QTLs can be combined by the inter-mating of particular (desirable) individuals followed by selection in each cycle to develop improved (HS tolerant) population via MARS approach [169]. Although a numerous QTLs were identified for HS tolerance in the last decade in different crops, as expected for complex traits, most of them were of minor effect, hence resulting in limited genetic gains in breeding HS tolerant cultivars. The MABC based introgression of major QTLs for abiotic stress tolerance has been reported in rice [170]. 

In wheat, elite cultivars HD 2733 and GW 322 varieties were targeted for introgression of identified QTLs for HS tolerance associated traits, namely canopy temperature depression, chlorophyll content, thousand kernel weight, and grain yield, via a foreground selection approach [169]. The background selection in progenies derived from two backcrosses revealed more than 90% recovery of the elite parent. The lines need to be further characterized for HS tolerance in multiple location trials [169]. The identification of critical genes controlling HS tolerance in common wheat led to the release of new cultivars of bread wheat and durum wheat capable of withstanding severe HS [171], e.g., the cultivar Faraj that is able to maintain yield under HS and drought conditions [172]. Wild relatives serve as a great reservoir of favorable alleles for abiotic stresses. In wheat, introgression of drought-resistant QTLs from wild emmer wheat to elite durum and bread wheat led to enhanced drought tolerance in introgressed progenies in drought stress ecology, hence providing a clue to explore wild relatives in HS breeding in wheat. 

Furthermore, the availability of cheaper sequencing platforms and advances in omics approaches will speed up molecular breeding for the identification and functional validation of candidate genes [173]. Furthermore, the rapidly evolving next-generation sequencing platforms have also assisted in providing highly dense markers that have enabled high-resolution mapping of QTLs in GWAS and GS studies [163]. Candidate genes for HS tolerance can be identified by positional cloning or a chromosome walking approach of major QTLs as well as transcriptome profiling of the HS tolerant and susceptible genotypes. Consensus QTLs can also be identified by conducting QTL mapping trials in optimum as well as stress environments. The introgression of identified consensus QTLs can lead to the development of climate-smart cultivars [174]. Furthermore, the selected HS tolerant cultivars need to be screened against other stresses, e.g., drought, waterlogging, and mineral deficiency, to develop multiple-stress tolerant cultivars. Although QTL mapping is a good approach for identifying HS tolerant genes, Meta-QTL analysis is more statistically powerful approach to pinpoint genomic regions as it is based on a meta-analysis of different individual studies for the concerned trait. 

QTL meta-analysis was carried out by using the data from 30 different studies for traits related to HS and drought stress tolerance [175]. The study revealed the presence of eight major meta-QTLs HS and drought tolerance associated traits on eight different chromosomes revealing the independent localization of genes for complex traits. Fine mapping of these regions can further help in the in-depth characterization of identified clusters [176]. Although, QTL mapping does not contribute as expected, the information can supplement the GWAS and GS approaches, as the use of these approaches is expected to be adopted at higher pace for the identification of novel SNPs due to the availability of cheaper genotyping platforms [175]. 

Valluru et al. (2016) conducted SNP based GWAS study in a panel of 130 diverse wheat germplasm for elucidating the possible role of ethylene hormone in spike development in bread wheat under stress conditions [177]. The study revealed a significant variation for the spike ethylene production in wheat germplasm, for which a total of 37 significant SNPs were identified under HS. Hence, wheat cultivars having the ability to produce reduced ethylene under HS have the potential to maintain better yields [177]. The GS approach is based on the utilization of genome-wide SNP marker information to build a genomic prediction model for the rapid and accurate selection of progenies. Hence, GS increases annual genetic gains from selection through the reduced frequency of phenotyping as well as cycle time. Since the discovery of the GS approach, HS breeding has gained momentum to identify the causal SNPs for HS tolerance associated traits. 

However, being a self-pollinated crop and relying upon the population selection in early segregating generations, wheat witnessed less use of GS as compared to other major crops because the majority of crosses are discarded at initial stages. Certain modifications in GS approach can make its utilization quite effective in wheat. The integration of recurrent mass selection with GS in advanced generations (F_3_ or F_4_ with high throughput phenotyping or phenotypic selection in preceding generations i.e., F_2_ and F_3_) is a relatively better approach for the introgression of HS adaptive genes from diverse germplasm, hence achieving further higher genetic gains for HS tolerance in wheat [178]. The completion of the annotated reference sequence of bread wheat genome revealed the presence of 107,891 high-confidence genes and is likely to speed up the HS resilience breeding [179]. Furthermore, the progress for the development of HS tolerant cultivars in wheat will be further accelerated by the adoption of a speed breeding technique that significantly fastens the generations under controlled conditions, hence making it possible to advance 5–6 generations per year [180]. 

#### 4.1.5. Epigenetics Approaches 

Besides the morphological and molecular level of adaptation, plants attain adaptation through epigenetics. Epigenetics, literally meaning “above genetics”, is the phenomenon of heritable changes via altered gene expression (expression level) instead of altered nucleotide sequences (genetic level). Epigenetic changes include alteration of gene/genome methylation pattern, histone-modifications, and non-protein coding RNAs for survival under abiotic stresses [181]. Epigenetics has a potential role in imparting HS tolerance, as evident from gained momentum for its utilization in HS resilience breeding [182]. In an epigenetics study, e.g., *GCN5* gene in *Arabidopsis*, histone acetyltransferase *TaGCN5* gene from wheat, when overexpressed in *Arabidopsis*, was found to impart HS tolerance through histone hyperacetylation [183]. The findings were further validated by examining the post HS promotors-targeted acetylation level of histones (*H3K9* and *H3K14*) in six heat-induced and unregulated genes, including *TaHSF1, TaHSF4, TaMBF1c, TaHSP17.4, TaHSP26*, and *TaHSP101*. The upregulated genes have enhanced expression under heat stress only, while unregulated genes (constitutive/housekeeping genes) show continuous expression irrelevant of heat or other type of stresses. [141]. The study agreed with earlier findings except for one gene [141]. 

However, another genome-wide expression-based study on hexaploid wheat exhibited contrasting results with minimum effects on DNA methylations patterns under HS [182]. These findings suggest the need to conduct more research in wheat for pinpointing the reason for the association of histone acetylation and DNA methylation in the regulation of HS responsive genes. Non-protein coding RNAs such as miRNAs also play vital role in regulation of HS-responsive genes in wheat [182]. In an experiment on identification of mi-RNAs involved in HS tolerance, Xin et al., (2010) discovered 12 HS responsive miRNAs [184]. Similarly, in another study, six novel HS responsive mi-RNAs were identified in wheat [185]. The differential expression of pre-miRNAs under HS in durum wheat cv Cappelli and Ofanto revealed the involvement of 12 and 25 miRNAs, respectively [141]. 

#### 4.1.6. Genetic Engineering and Functional Genes 

Genetic engineering or transgenic breeding is an alternative strategy for the development of HS tolerant wheat cultivars [141]. Transgenic breeding also addresses the problem of linkage drag. In addition, the variation for a target trait not existing in a particular species can also be introduced de novo from an unrelated organism by genetic engineering. However, the relatively complex genomic pattern in wheat has hampered the progress of HS tolerance breeding [186]. HS induces the higher buildup of elongation factor (*EF-Tu*), involved in protein synthesis in wheat chloroplast [187,188]. The overexpression of maize *EF-Tu1* gene in transgenic wheat imparted HS tolerance due to relatively better photosynthesis capacity, stability of thylakoid membranes, safeguarding against denaturation of leaf proteins and pathogen resistance over non-transgenic wheat plants [2,141]. The overexpression of maize phospho-enol-pyruvate carboxylase gene (*ZmPEPC*) in wheat is also involved in imparting HS tolerance via the enhanced activity of photochemical and antioxidant enzymes, prolonged retention of chlorophyll content, altered proline accumulation, and upregulation of photosynthetic machinery regulating genes. Hence, transcriptional factors/candidate genes play vital role in imparting HS tolerance to wheat as evident from their successful genetic engineering mediated transfer and expression in different crops [189,190]. The improved sequencing technologies facilitate the assembly and annotation of bread wheat genome. Hence, the contrasting varieties for HS tolerance can be used to identify the key chromosomal arrangements, imparting wide adaptation of wheat cultivars under HS ecology [2].

Functional genes that respond well under HS in wheat have already characterized in rice or *Arabidopsis* (Table 5). Examples of such functional genes in wheat are *TaHSFA6f*, *TaFER-5B*, and *TaPEPKR2* that impart thermo-tolerance through overexpression under HS [191]. Besides these examples, a gene from *Arabidopsis, AtWRKY30* was tested in the wheat background for expression under HS and drought stress. The gene was found to be overexpressed under stress conditions [190]. The compilation of candidate genes of bread wheat that exhibit over-expression under HS in plants are provided in Table 5. Although lot of progress has been achieved for genetic-engineering mediated introgression of HS responsive functional genes in wheat, the genetic background effect for these genes remains to be explored. However, the recent advances in transformation technology and accessibility to mutant libraries of wheat will speed up the functional characterization of HS responsive genes [192].

### 4.2. The Interplay of Omics Approaches in Adaptation to HS

**Figure 3:**
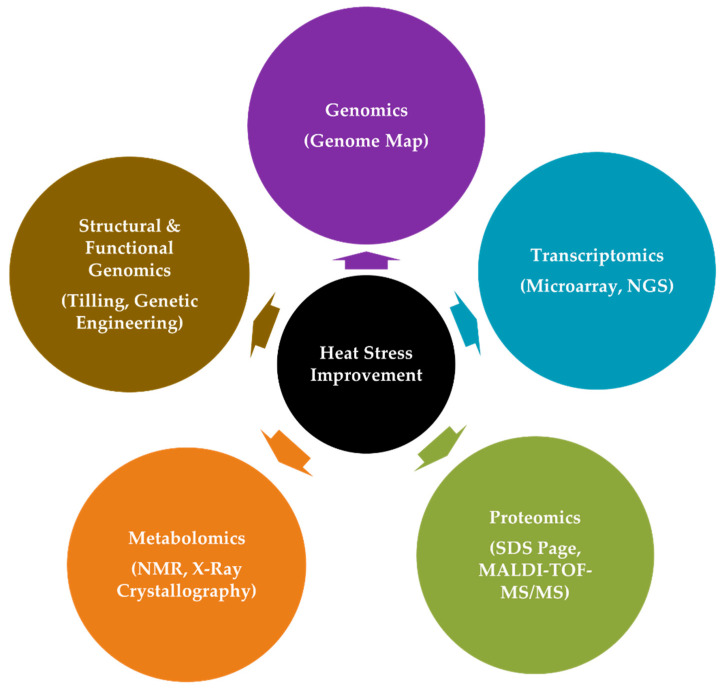
Different omics-based approaches for inculcating HS tolerance in wheat.

Omics play an important role in the identification of the differentially expressed genes/protein/metabolites for heat stress response in contrasting cultivars (tolerant and sensitive). Omics approaches, e.g., transcriptomics, proteomics, and metabolomics, help to understand the adaptation or tolerance mechanism through an investigation of the key genes/enzymes/metabolites regulating the key pathways for heat stress response in crops. Furthermore, phenomics is a promising approach for precise high throughput phenotyping of the heat stress associated traits. Phenomics is more or less becoming an integral part of the genomic selection and genome wide association studies in different crops. The omics approaches help to understand the regulatory network of genes and the interplay therein responsible for plant tolerance to temperature stresses, *viz*. heat, cold, and frost. Plant response to HS depends primarily on up-regulation and down-regulations of different HSP family of genes. Besides, having a detailed theoretical knowledge, an in-depth genomics study is needed for a widespread understanding at the molecular level. Further, an integrated omics-based approach, drawing on genomics, transcriptomics, proteomics, and metabolomics, will help in the identification of key target candidate genes regulating the photosynthesis, osmoprotectants, and antioxidant compounds and in the deeper understanding of HS tolerance mechanism of different wheat cultivars (Figure 3). Several online omics-based in-silico tools, e.g., WoLFPSORT, CELLO server.2.5, and MEME suite, and databases, such as TIGR Genome Database, HarvEST Wheat, ArrayExpress, and PLEXdb, provide a suitable platform for comprehensive study on plant HS combined with wheat genome engineering using CRISPR/Cas System. 

#### 4.2.1. Genomics

The genome is the sum total of genetic make-up of an organism and genomics provide in-depth information concerning gene structures, functions, interconnected networks, as well as metabolic and biochemical processes they are involved in [202]. Genomics evolved in diverse stages from first generation (1970s) to next generation (20th Century) to third generation sequencing (21st Century) [203]. Structural genomics helps to identify structure of a gene, prompter sequences, and other regulatory sequences located upstream or downstream [204], whereas functional genomics facilitate gene function at a molecular level and the type of tolerance (HS, salt, drought) it provides [205,206]. Therefore, genomics aids in providing genome wide network information of genes and their interaction with other complex stress-resilient traits [207]. Wheat cultivars can be genetically engineered for HS tolerance using the CRISPR/Cas9 system and other online genomics tools for further research and understanding on plant abiotic stress tolerance [208]. The fully annotated complete reference genome of wheat (cultivar ‘Chinese Spring’) was completed in 2018 and is available at Unité de Recherche Génomique Info (URGI) research unit in genomics and bioinformatics at French National Institute for Agriculture, Food and Environment (INRAE) (https://wheat-urgi.versailles.inra.fr/Seq-Repository; (accessed on 16 February 2022) and International Wheat Genome Sequencing Consortium hosted at ENSEMBL-Plants portal (https://plants.ensembl.org/Triticum_aestivum/Info/Index; accessed on: 16 February 2022) [179].

#### 4.2.2. CRISPR/Cas9: The Promising Future

**Figure 4:**
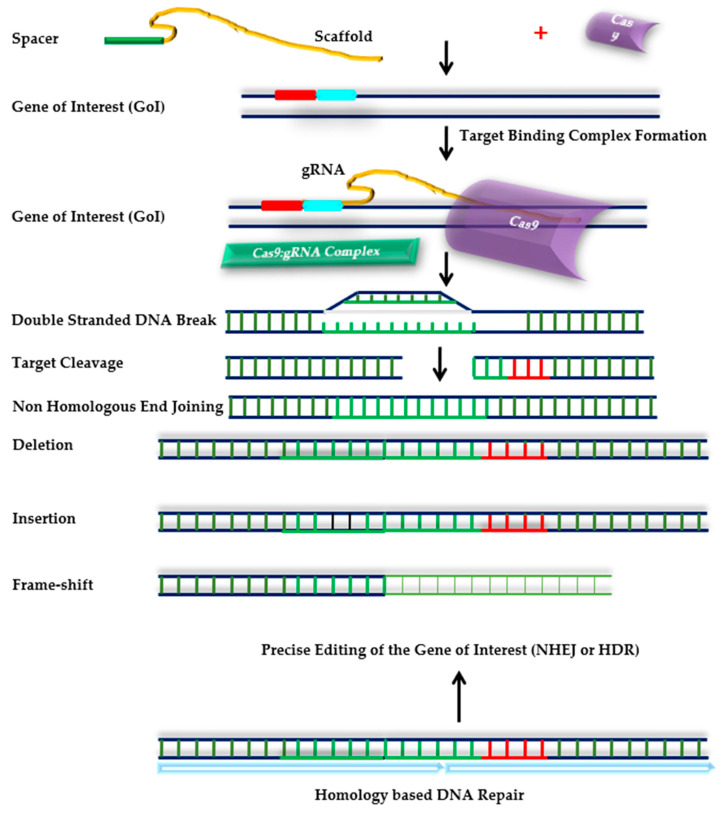
Flow chart of Crispr-Cas9 homology based precise genome/DNA editing for developing HS resilient wheat cultivars.

CRIPSR/Cas9 is a unique and proficient DNA editing tool used throughout the world due to its broad array of applications in both prokaryotes as well as eukaryotes (plants and animals) [209]. It revolutionized the functional research in conventional plant breeding and genetics and is successfully employed to edit genome of major crops for diverse abiotic stresses, *viz*. heat, drought, and salt. CRISPR-Cas9 edits the target gene via deletion, insertion, and knock-in/knock-out mutations, thus improving crop plants for their reactive oxygen species (ROS) scavenging ability [210] (Figure 4). This homology-based genome editing tool has been thoroughly used to improve temperature tolerance in diverse plant species [209,210,211]. The system has identified a target gene, *OsNTL3*, responsible for providing HS tolerance to rice crop, which encodes for a NAC transcription factor and switches on the regulatory circuit of nucleus, endoplasmic reticulum, and cellular membrane [211]. Similarly, in wheat, Tian et al. [212] applied a combined approach of CRISPR/Cas9-based gene editing, polysome profiling, RNA-sequencing analysis, and protein–protein interactions to reveal that *TaMBF1c* (upregulated under heat stress) is an important gene that imparts heat response in wheat via regulating the translation process [212].

#### 4.2.3. Transcriptomics

A transcript is an expressed part of a genome, and studying transcripts with different technological advancements, *viz*. RT qPCR, micro array, and next generation sequencing, is known as transcriptomics [213]. Recently, Arenas-M et al. [214] identified the role of novel TFs, e.g., members of the DOF family, that impart better grain quality via regulating glycogen and starch biosynthetic processes in grains under short term heat stress. This helps in discovering underlying molecular and physiological mechanisms of plant response to different abiotic stresses. The location and abundance of a transcript at a particular cellular stage of growth and development provides better understanding under different abiotic stresses, particularly, HS. Transcriptome profile under HS (>42 °C) has been studied primarily for rice, which identified different clusters of deferentially expressed genes (DEGs) involved in signal transduction and photosynthesis [215]. By RNA Seq analysis, 50 DEGs were identified in wheat under HS of flag leaf and root and specifically targeted towards stress and cell cycle, protein processing, starch and sucrose metabolism, secondary metabolite biosynthesis, photosynthetic transport and other catabolic processes [136]. In another study by Ma et al. [216] considering the transcript profile of wheat under drought stress, 37 up-regulated DEGs were found and were primarily engaged in “C” fixation, Mg^+2^ binding, and ribulose bisphosphate carboxylase activity. Later, Rangan et al. [119] reported genes, namely 6-phosphogluconate dehydrogenase, S6 RPS6-2 ribosomal protein, peptidylprolyl isomerase, plasma membrane proton ATPase, Heat shock cognate-70, FtsH protease, RuBisCO activase B, methionine synthase, cytochrome C (class I), and HMW-glutenin, as key candidate genes for heat stress tolerance during grain filling. Therefore, transcriptome profiling can precisely provide detailed insight into the sophisticated molecular and biochemical mechanisms regulating plant response to unusual stresses. Conversely, only limited research has been carried out on the wheat response to HS at different stages of growth and development [217,218].

#### 4.2.4. Proteomics

Proteins are expressed parts of a genome and studying them using several tools, *viz*. MALDI-TOF/MS, 2D PAGE, SDS PAGE, and HPLC, provides information about their role in special abiotic stress. It provides knowledge about specific proteins role in the abiotic stress signal transduction pathway [65]. HS affects critical proteins involved in cellular metabolism, photosynthesis, replication, transcription, and translation. Proteomic analysis under HS has been conducted on most of cereals crops, e.g., wheat [219,220], rice [221], and barley (*Hordeum vulgare*; [222]) and several other crops, e.g., tobacco (*Nicotiana tabacum*; [223] and mustard (*Brassica juncea*; [224]). These proteins were associated with osmolytes, electrolytes, hormonal signaling, leaf senescence, and other cellular homeostasis, as shown in wheat [67] and potato [225]. Signaling molecules (MAPKs and CDPKs), HSPs (HSP17, HSP20, HSP26, and HSC70), and antioxidant enzymes (SOD, CAT, and APX) were found to be the key proteins imparting heat stress tolerance to the organelles. However, knowledge gaps remain concerning the role of specific proteins providing HS tolerance to different wheat cultivars and the different response of sensitive and resistant cultivars. Therefore, there is a need for further research on proteome analysis in wheat under HS tolerance.

#### 4.2.5. Metabolomics

Metabolome is total of metabolites providing plant response to specific stressed condition. It determines gene and transcript expression, protein buildup and their post translational modification. HS affects different biochemical pathways, *viz*. glycolysis, citric acid cycle, and electron transport pathway and the accumulation of antioxidant enzymes. The wheat plant synthesizes an array of metabolites that differentially varies with the stress conditions. The difference in the metabolite expression correlates with different genotypic and phenotypic traits and makes it a powerful tool for the selection of the heat-tolerant cultivars which have association with the phenotype and genotype interactions. Metabolites affect phenotypes to a greater extent and therefore need to be identified while studying plant response to abiotic stress tolerance. Metabolomics is done by X-ray crystallography, GC-MS, and TOF-MS, providing a novel understanding of plant response to HS by detecting metabolic patterns [210]. Metabolomics of several crops has been studied under HS, in wheat seeds [218], *Arabidopsis* leaves [215], rice immature seeds [226], and *Populus tomentosa* leaves [227]. In wheat, lower amounts of phospholipids with oxidized acyl chains and higher amounts of sterol glycosides and 16:0-acylated sterol glycosides has been reported to confer heat stress tolerance under high day and night temperature [80]. However, little is known about the metabolome of wheat under different levels of HS, i.e., prolonged and heat shock, as metabolites serve as potential bio-markers for genetic improvement. Studies on wheat revealed that metabolites such as L-tryptophan and pipecolate under heat stress were reported to be enhanced. The same study suggested that metabolites, such as drummondol and anthranilate, were reported to be significantly reduced under heat stress. These results showed that the metabolites synthesis is highly regulated by the environmental factor and affects phenotypic expression in the wheat plant [228]. Moreover, the source–sink relationship under HS needs to be established for different wheat cultivars, which may give valuable insight into improving the protein content of wheat. Therefore, by engaging genetic and breeding approaches with modern biotechnological tools, innovative strategies can be designed to enhance wheat grain quality under HS environment [93].

### 4.3. Agronomic Strategies

#### 4.3.1. Efficient Nutrient Management

Efficient nutrient management is one way to reduce the impact of HS and sustain crop productivity. Optimization of nitrogen (N) supply sustained the wheat yield by increasing the stomatal conductance, chlorophyll contents and photosynthetic rate under elevated temperature [38,229]. The application of nitric oxide form of N protects the plant from oxidative damage by functioning as scavenger of ROS. Combined application of thiourea as seed treatment and foliar spray was more effective in improving heat stress tolerance of wheat by enhancing membrane stability, antioxidant potential [230]. Under terminal heat stress condition, foliar application of K in the form of potassium orthophosphate (KH_2_PO_4_) helps to activate the various physiological and metabolic processes, such as photosynthesis, respiration, and nutrient homeostasis, and it increases the tissue water potentiality, which assists in extreme temperature stress tolerance. Likewise, foliar application of silicon at the heading stage alleviates the negative impact of terminal heat stress by improving the antioxidant mechanism and production of osmoprotectants [230]. The application of calcium (Ca) in the form of CaCl_2_ and CaNO_3_ promotes heat tolerance by improving rate of photosynthesis and activate antioxidant enzymes and increased amino acid contents [231]. Magnesium (Mg) and sulfur (S) deficiency in wheat increases susceptibility to HS and hence ensured optimum supply through fertilization can minimize the HS related losses [232]. Optimum supply of Zn through foliar fertilization can maintain membrane integrity and activity of SOD enzymes [233] thus imparting HS resilience in wheat. Similarly, foliar application of boron (B) at flowering stage of wheat crop has also been reported to improve HS tolerance by increasing antioxidant activities and alleviating accumulated ROS [234,235].

#### 4.3.2. Use of Plant Growth Regulators

Another approach to alleviate HS in crops is the use of exogenous osmoprotectants either as seed inoculation or foliar application. Some osmoprotectants, such as inorganic salts, stress signaling molecules, nitrogenous compounds, and synthetic and non-synthetic plant growth regulators, have the potential to improve photosynthetic rate and physiological function of wheat under HS [236]. For example, the application of selenium (Se) in sorghum improves the osmotic adjustment capability, activity of antioxidant and chlorophyll content [42,237]. This reduces the chances of oxidative damage by ROS and electrolyte leakage under HS condition [4]. Likewise, foliar spray of salicylic acid (SA) at the grain filling stage of wheat helped to increase the HS tolerance capacity through lowering the photoinhibition by maintaining higher photochemical activity of PS-II, chlorophyll content and net photosynthetic rate [238]. The foliar application of progesterone also protects the wheat against HS condition by reducing oxidative damage and improving the photochemical efficiency of PS-II and oxygen-evolving activity under HS [239]. Similarly, the application of CaCl_2_ also improved the HS tolerance capacity and biomass in wheat by enhancing the nitrate reductase activity and RLWC [230] Furthermore, the application of silver nanoparticles and some of the plant growth regulators, e.g., ABA, indole acetic acid (IAA), and Gibberellic acid (GA), have been reported to increase the grain yield of wheat by improving the grain number and weight under elevated temperature [240].

#### 4.3.3. Use of Cultured Soil Microbes

Some cultured soil microbes, such as plant growth promoting Rhizobacteria (PGPR) and arbuscular mycorrhizal fungi (AMF), have the potential for improving HS tolerance in plants. Therefore, the use of these microbial inoculants either as seed treatment or seed priming has been considered as an alternative eco-friendly approach [241]. For example, seed inoculation with some PGPR strains, such as *Bacillus amyloliquefaciens* (UCMB5113) and *Azospirillum brasilense* (NO40), improved HS tolerance in wheat seedlings by reducing the oxidative damage through arresting the production of ROS under elevated temperature [242]. Likewise, *Pseudomonas putida* strain AKMP7 has also been reported to improve the survival and growth of wheat under HS. Inoculations with AKMP7 strain significantly increased the root and shoot length, level of cellular metabolites like proline, chlorophyll, sugars, starch, and amino acids and reduced membrane injury by improving the activity of several antioxidant enzymes, such as SOD, APX, and CAT, under HS [243]. AMF symbiosis in wheat is a compatible mechanism that imparts a beneficial effect on the growth and development of wheat under HS as it decreases the potassium to calcium ratio and increases availability of photosynthates in the spikes and improves grain number. Seed treatment endophytic fungal strain SMCD 2215 has been reported to decrease the adverse effect of HS in wheat by improving germination and neutralizing the oxidative damage by reducing the pace of ROS production [244]. However, more investigations, especially through field trials, are further required to confirm the potential heat tolerance mechanisms by which these PGPRs and AMF confer benefits to their hosts.

#### 4.3.4. Modification in Planting Time and Method 

The adverse impact of HS may be reduced by modifications to the sowing time and method. The change in the time of sowing of wheat is a vital non-monetary management input to cope with or escape HS. In general, late sown wheat is more likely to face HS at time of post-flowering or early grain filling (terminal HS), resulting in a reduced test weight of grains due to impaired grain filling [245]. Therefore, early sowing is essential to avoid terminal HS and the adoption of CA provides the avenue for advancing the sowing of wheat by 15–20 days in North-Western India [246]. Moderation of soil and canopy temperature by retaining crop residue on soil surface with the adoption of conservation agriculture (CA) based management practices is considered the best strategy to alleviate the negative impact of HS. The adoption of CA-based management strategies, such as zero tillage (ZT), permanent bed planting (PBP), and furrow irrigated raised bed systems (FIRB), in wheat can reduce the canopy temperature by 1.5–3.0 °C depending on the residue load crop type in the cropping system and biomass production capacity genotypes used. Ensuring early or timely sowing (avoiding delay sowing), especially in the rice-wheat cropping system of India, with the adoption of CA practices could be considered an ideal strategy to obtain an optimum yield under HS-prone ecology [247].

#### 4.3.5. Precise Irrigation and Soil Moisture Conservation

Water management is crucial to avoid the adverse impact of HS on crops, especially in the rainfed wheat-growing ecology of the world. As terminal HS is often accompanied by terminal drought stress, irrigation techniques, such as sprinkler and drip, which focuses on the increasing frequency of irrigation, could be a better option for mitigating the impact of terminal HS and drought stress. Irrigation through sprinkler systems may help to lower the soil as well as canopy temperature (optimizes micro-climate) and decrease the vapour pressure deficit. Similarly, drip irrigation also helps to maintain adequate moisture in soil and reduces canopy temperature by optimizing the transpiration process [248]. Besides, mulching is an effective approach to maintain the soil moisture and temperature at the optimal level and hence improves biomass production, particularly under rainfed conditions. Mulching on the soil surface helps to maintain soil moisture, resist fluctuations of soil temperature, and improves soil aeration, which ultimately enhances the emergence of seedling and root growth [249]. In addition, the availability of soil moisture can help wheat plants to minimize canopy temperature through transpiration cooling and avoid HS.

## 5. Conclusions

HS mediated changes in biological and developmental processes of wheat adversely affect the yield and quality. HS affects physio-biochemical and molecular processes, such as emergence, photosynthesis, respiration, nutrient-water relations, membrane stability, stem reserve accumulation, photo-assimilates partitioning, reproduction, and grain development of wheat. Hence, a systematic phenome to genome analysis is required for precise trait mapping, introgression/incorporation/enrichment of superior alleles, or gene cloning for HS tolerance. Such analysis can help to identify genes responsible for HS tolerance by the establishment of precise relationships between phenotypes and genotypes. Efficient utilization of known HS tolerance mechanisms and candidate genes can provide new clues for tailoring wheat genotypes that generate optimum yields under HS environment. Genetic engineering and biotechnological tools should be integrated under the wheat breeding programs for confirmation of HS related candidate genes. A blend of different omics approaches along with speed breeding will provide a new window of opportunity to develop HS tolerant wheat varieties. A deeper understanding of wheat-omics will provide information about novel marker genes and their further utilization for breeding purpose. Therefore, genetic engineering and molecular breeding tools (QTLs/candidate gens, MAS, and epigenetics) should be combined to achieve better HS tolerance. Some of the agronomic management strategies, such as the application of exogenous protectants, adoption of climate smart cultivation practices (CA, micro-irrigation, and mulching), and use of cultured soil microbes, are promising in increasing the resilience of wheat to HS. However, the complex nature of HS tolerance demands a multidisciplinary, holistic approach integrating the outcomes of physiological, breeding, and agronomic management options to sustain wheat production under current and future changing climates.

## Figures and Tables

**Table 1:** Optimal temperature requirements of wheat at different growth stages (Adopted from Khan et al. [20]).

Stages	Optimum Temperature (°C)	Minimum Temperature (°C)	Maximum Temperature (°C)
Seed germination	20–25 ± 1.2	3.5–5.5 ± 0.44	35 ± 1.02
Root growth	17.2 ± 0.87	3.50 ± 0.73	24.0 ± 1.21
Shoot growth	18.5 ± 1.90	4.50 ± 0.76	20.1 ± 0.64
Leaf initiation	20.5 ± 1.25	1.50 ± 0.52	23.5 ± 0.95
Terminal spikelet	16.0 ± 2.30	2.50 ± 0.49	20.0 ± 1.60
Anthesis	23.0 ± 1.75	10.0 ± 1.12	26.0 ± 1.01
Grain filling duration	26.0 ± 1.53	13.0 ± 1.45	30.0 ± 2.13

**Table 2:** Impact of heat stress on different traits/biological processes of wheat.

Trait/ Biological Process	Responses/Consequences/Impact	References
Morpho-phenological behavior	Poor germination and seedling establishment	[34,40]
	Reduction in root length, shoot growth and dry matter	[34,41]
	Reduction in effective tiller	[42]
	Reduced ear length, number of spikelet and fertile floret	[43,44]
	Abortion of flower and fruits	[11,12,41]
	Shedding of leaves	[45,46]
	Reduction in phenological duration of crop	[14,47]
	Reduced days to germination, anthesis and maturity	[14,43]
	Reduction in germination of pollen grains and spikelet fertility	[11,12,48,49]
	Reduced grain filling period	[11,14,43,45]
Grain development and quality	Reduction in number or size of grain	[11,14,19]
	Reduction in harvesting index	[48]
	Increases rate of grain filling but shortened grain filling duration	[50]
	Reduction in transportation of photo-assimilates to grain	[51]
	Increase in grain protein and reduction in quality of proteins	[52]
	Reduced starch synthesis	[51,52]
	Reduced total soluble sugar and super molecules	[51,53]
	Reduction in essential amino acids	[50]
	Reduced bread making quality	[52]
	Reduction in flour quality and sedimentation index	[50,54]
	Reduction in economic grain yield	[51,55]
Physiological and growth behavior	Reduced photosynthesis and photosynthetic efficiency	[14,18,19,56,57]
	Increase in respiration and photorespiration at mild heat stress	[56,57]
	Increase in leaf senescence and reduction of chlorophyll content	[14,18,19,39,46]
	Reduction in the relative water content and leaf water potential	[45,58]
	Increased transpiration and decreased stomatal conductance	[59]
	Decrease uptake and translocation of water	[50,60]
	Increased canopy temperature	[61]
	Reduction in uptake, assimilation, and translocation of nutrient	[62,63]
	Reduction in specific leaf weight, leaf width and total dry matter	[38,64]
Molecular responses	Enhanced production of reactive oxygen species (ROS)	[18,19,65,66]
	Higher accumulation of osmolytes	[36,65]
	Destruction of plasma, mitochondrial and chloroplast membrane	[34]
	Reduction in Rubisco activity	[66]
	Reduction in soluble and rubisco binding proteins	[16]
	Denaturation and aggregation of seed proteins	[67]
	Higher accumulation of heat shock proteins	[68,69]
	Activation of antioxidant system and associated molecules	[70,71]

**Table 3:** ROS scavenging reactions by different antioxidants at different cellular sites (Compiled from Blokhina et al., 2003; Ashraf, 2009; Gill and Tuteja, 2010) [112,113,114].

Antioxidants	Major Catalyzed Reactions	Site of Reactions
Super Oxide Dismutase (SOD)	2O_2_^−^ + 2H^+^ → H_2_O_2_ + O_2_	Chlorophyll, Cytosol, Apoplast, Mitochondria, Peroxisome
Catalse (CAT)	H_2_O_2_ → H_2_O + ½O_2_	Peroxisome, Chlorophyll and Mitochondria
Ascorbate peroxidase (APX)	H_2_O_2_ + 2AsA → 2H_2_O + 2MDHA	Chlorophyll, Cytosol, Apoplast, Mitochondria, Peroxisome
Monodehydro ascorbate reductase (MDHAR)	NADPH + H^+^ + 2MDHA → 2AsA + NADP^+^	Chlorophyll, Cytosol and Mitochondria
Dehydroascorbate reductase (DHAR)	DHA + 2GSH → AsA + GSSG	Chlorophyll, Cytosol and Mitochondria
Glutathione reductase (GR)	NADPH + H^+^ + GSSG → 2GSH + NADP^+^	Chlorophyll, Mitochondria and Cytosol
Glutathione peroxidase (GPX)	2GSH + ROOH (H_2_O_2_) → GSSG + ROH + H_2_O (2H_2_O)	Mitochondria and Cytosol
Glutathione -S-transferase (GST)	H_2_O_2_ + 2GSH → 2H_2_O + GSSG RX + GSH→ HX + GS-R	Chlorophyll, Cytosol and Mitochondria
Ascorbate (AsA)	Scavenges O_2_^−^, H_2_O_2_, OH^·^, and O_2_	Chlorophyll, Cytosol, Apoplast, Mitochondria, Peroxisome
Glutathione (GSH)	Scavenges H_2_O_2_, OH^·^, and O_2_	Chlorophyll, Cytosol, Apoplast, Mitochondria, Peroxisome
Tocopherol	Scavenges O_2_, OH^·^, ROO^·^ and ROOH	Membranes

**Table 4:** List of recently mapped major QTLs for heat stress resistance in wheat.

Trait	QTLs	Mapping Population	Cross	Chr.	Markers	PVE (%)	References
Grain Yield	*QYld.aww-1B.2*	DH	Excalibur×Kukri	1B	*adw1218477-BS00022342*	>15.0	[153]
Thousand-grain weight	*QTGW-2A.1*	RIL	SYN-D (Croc1/ *A.* *squarrosa* (224)/ Opata) × Weebill 1	2A	DArTSeq	33.0	[154]
Grain yield per plant	*QGYPHS1*	DH	Hanxuan10 × Lumai 14	1B	*AX-111125138* *AX-95194017*	22.5	[155]
Fv/Fm (maximum quantum efficiency of photosystem II)	*QHst.cph-3B.1* *QHst.cph-3B.2*	F_2_	810 (IPK-2845) × 1110(IPK-9705)	3B	*Xgpw8020-1061426s* *1218388s-Xgwm389*	22.1 25.0	[156]
Maximum Fluorescence (Fm)	*QFm.cgb-4A*	DH	Hanxuan 10 × Lumai 14	4A	*Xwmc89-Xwmc420*	~15.0	[157]
Thylakoid membrane damage	*QHttmd.ksu-7A*	RILs	Ventnor × Karl 92	7A	*Xbarc121-barc49*	30.6	[158]
SPAD chlorophyll content	*QHtscc.ksu-7A*	RILs	Ventnor × Karl 92	7A	*Xbarc121-barc49*	30.8	[158]
Grain yield	*Q.Yld.aww-3B-2*	DHs	RAC875 × Kukri	3B	DArT and SSR	22.0	[159]
Heat susceptibility index (HSI) of 1000 grain weight	*QHthsitgw.bhu-7B*	RILs	NW1014 × HUW468	7B	*Xgwm1025* *Xgwm745*	20.3	[63]
HSI of kernel number	*QHknm.tam-2B*	RILs	Halberd × Cutter	2B	*Gwm111.2*	17.0	[160]

QTL, quantitative trait locus; DH, double haploid; RIL, recombinant inbred lines; Chr, chromosome; PVE, phenotypic variation explained.

**Table 5:** Potential candidate genes of bread wheat for heat resistance in plants.

Gene	Trans-Host	Function	Reference
*TamiR159*	*Oryza sativa*	Plants with *TamiR159* over-expressing gene were relatively more sensitive to high temperature environments than wild type plants	[193]
*HSP26*	*Arabidopsis*	Transgenic plants with *HSP26* gene more tolerant relative to the wild type under heat stress	[120]
*TaHsfA2d*	*Arabidopsis*	Transgenic plants with overexpressing gene *TaHsfA2d* showed improved level of tolerance	[194]
*TaHSF3*	*Arabidopsis*	Transgenic Arabidopsis with *TaHSF3* enhanced resistance under heat stress conditions	[195]
*TaLTP3*	*Arabidopsis*	TaLTP3-overexpressing plants showed relatively higher thermo-resistance under heat stress at the seedling stage than normal plants	[196]
*TaHsfA6f*	*T. aestivum*	Overexpression of gene *TaHsfA6f* in transgenic plants showed high thermo-resistance under extreme temperatures than control plants	[86]
*TaMBF1c*	*Oryza sativa*	Overexpression of gene *TaMBF1c* in plants showed improved thermo-resistance at both seedling and reproductive stages than normal plants	[197]
*TaNAC2L*	*Arabidopsis*	*TaNAC2L* genes in plants overexpressed to increase heat resistance by activating expression of heat-related genes under heat stress	[198]
*TaWRKY33*	*Arabidopsis*	Transgenic lines with *TaWRKY33* gene showed enhanced resistance to extreme heat stress	[199]
*TaB2*	*Arabidopsis*	Arabidopsis plants with *TaB2* gene overexpressed to exhibit resistance under heat stress	[90]
*TaHsfC2a*	*T. aestivum*	Overexpression of gene *TaHsfC2a* imparted resistance to heat stress	[200]
*TaFER-5B*	*T. aestivum*	Transgenic plants with *TaFER-5B* gene exhibited enhanced level of resistance under heat stress	[201]
*TaOEP16-2-5B*	*Arabidopsis*	Transgenic plants overexpressing the *TaOEP16-2-5B* gene exhibited improved thermo-resistance to stress environments	[201]
*TaGASR1*	*T. aestivum* or *Arabidopsis*	Transgenic plants with *TaGASR1* gene overexpressed to improve thermo-resistance under stress and resistance to oxidative stress also	[201]
*TaPEPKR2*	*T. aestivum* or *Arabidopsis*	Wheat gene *TaPEPKR2* transformed into the *Arabidopsis* or wheat cultivar Liaochun10 and transgenic lines exhibited enhanced heat and dehydration stress resistance.	[191]

## Data Availability

All data and materials are available upon reasonable request from the corresponding author.
